# Effect of Mechanical Vibration on the Mechanical Properties and Solidification Feeding in Low-Pressure Sand Casting of Al-Cu-Mn-Ti Alloy

**DOI:** 10.3390/ma15228243

**Published:** 2022-11-20

**Authors:** Wei Chen, Shiping Wu, Rujia Wang

**Affiliations:** School of Materials Science and Engineering, Harbin Institute of Technology, Harbin 150001, China

**Keywords:** mechanical vibration, Al-Cu-Mn-Ti alloy, LPSC, solidification feeding, mechanical property

## Abstract

The shrinkage defects of Al-Cu-Mn-Ti alloy seriously hinder its application in high-performance engineering. In this study, mechanical vibration was introduced to low-pressure sand casting (LPSC) by a waveguide rod to eliminate shrinkage defects and improve mechanical properties. Four LPSC castings were performed by changing the solidification conditions: 20 kPa solidification pressure without and with 14 Hz vibration and 40 kPa without and with 24 Hz (the natural frequency of a casting system) vibration. The shrinkage defects, microstructures, and mechanical tensile properties at room temperature and at 2 mm/min tensile rate were investigated. X-ray detections showed that applying vibration was more effective than increasing solidification pressure in improving solidification feeding, while the most effective method was applying both simultaneously, which eliminated the shrinkage defects and increased the density by 2.7%. Microstructures exhibited that the average size of primary *α*-Al grains were reduced by 29.5%; mechanical tests showed that the ultimate tensile strength and the elongation increased by 21.7% and 7.8%, respectively, by applying vibration and increasing the solidification pressure simultaneously, as compared to the casting with 20 kPa solidification pressure without vibration. Mechanical vibration was conducive to mass feeding by refining the primary grains, to interdendritic feeding by reducing the threshold pressure gradient, and to burst feeding by collapsing the barrier.

## 1. Introduction

Al-Cu-Mn-Ti alloy is widely utilized in military, aerospace, and aircraft industries for its light weight and superior corrosion resistance as well as its high specific strength after solution, quenching, and age hardening treatments [1]. The first precipitated TiAl_3_, of which the lattice orientation matches *α*-Al, is an appropriate heterogeneous nucleation core, which not only refines the matrix obviously but also contributes to the formation of equiaxed crystals. The secondary *T* (Al_12_CuMn_2_) phase dispersed within the matrix enhances the strength of this alloy greatly. After an aging treatment, the highest ultimate tensile strength can be improved to 488.2 MPa [2]. Therefore, various manufacturing processes of Al-Cu-Mn-Ti alloy have been widely studied [3,4], in which the casting is the most common technology [5]. However, due to its wide crystallization temperature range, the casting of the alloy shows a wide, mushy region in solidification, magnifying the feeding resistance, which increases the occurrence of shrinkage defects [6], especially in large, thin-walled castings [7] where a series of shrinkage defects are observed in the macrostructure and microstructure [8]. The shrinkage defects can cause serious adverse effects on the performance of castings, which is difficult to eliminate by a subsequent thermal–mechanical treatment or forging process [9]. Therefore, it is necessary to eradicate shrinkage defects in the solidification process.

A traditional way to eliminate shrinkage defects is to realize a sequential solidification by improving the gating system [10], rationally setting risers and chill irons [11], altering the casting modes [12], and regulating the melt condition [13]. However, the traditional methods will increase the complexity of the solidification process or cause secondary pollution to the alloy melt, and sometimes they cannot obtain an obvious improvement in some large, complex castings [9]. Fortunately, the casting feeding capacity can also be improved by adding an external force to the existing casting system. To determine the form of external force, two aspects should be considered: increasing a feeding pressure and dredging a feeding channel [14]. Increasing the feeding pressure is a common method to improve the feeding capacity, such as increasing the holding pressure in the low-pressure casting and increasing the rotational speed in the centrifugal casting. However, when the casting structure is complex or the feeding channel is long, increasing the feeding pressure cannot obtain an obvious improvement because the feeding channel is easy to be blocked by solidifying grains. As an example, the porosity defects were formed in the large, thin-walled cylindrical casting of the Al-Cu-Mn-Ti alloy, despite increasing the solidification pressure [15]. Accordingly, it should be more effective to dredge the feeding channel while increasing the feeding pressure. Regrettably, research to date has been more focused on increasing the feeding pressure, with little attention on dredging the feeding channel. 

Mechanical vibration, as an excellent plugging removal technology, is widely used in oil exploitation [16] and also has been applied in casting to ameliorate solidification [17,18,19]. Yadav [20] and Jiang et al. [21] compared the defects and porosities of a cast aluminum alloy without and with vibration and found that vibration reduces the shrinkage porosities and increases the mechanical properties of samples. Pîrvulescu et al. [22] applied a 50 Hz mechanical vibration to gravity aluminum castings and detected that vibration can effectively degas the melt and transform the dispersive microporosities to the concentrated shrinkage cavity. Kocatepe et al. [23] investigated that the pipe in the ingot is reduced by vibration with an increasing frequency, amplitude, and vibration time of the liquid. Zhao et al. [24] concluded that vibration facilitates the diffusion of hydrogen atoms, improving the hydrogen evolution, thus reducing the shrinkage porosities and the pinhole ratio on the section. Chen et al. [25] studied the effect of mechanical vibration on the sand gravity casting of Al-5%Cu-0.4%Mn alloy and found that vibration can influence the interdendritic feeding. Therefore, mechanical vibration may be an effective method to dredge the feeding channel. However, up to now, mechanical vibration has been mainly used in gravity casting [26], but its effect on the low-pressure casting process is rarely studied. The effect and mechanism of mechanical vibration on the mechanical properties and solidification feeding in low-pressure sand casting (LPSC) is not clear. Therefore, in this work, mechanical vibration was introduced into the LPSC of Al-Cu-Mn-Ti alloy.

Mold oscillation is a main introduction method of mechanical vibration, requiring a platform with vibrating motors [20,21,22,23,24,25,26]. Because a casting system is heavy in the large casting and the LPSC, high-power motors are needed to generate significant vibrations; so, mold oscillation is only suitable for the production of small- and medium-sized castings. Meanwhile, mold oscillation causes hidden danger to the seal of the LPSC process. As the purpose of applying vibration is to dredge the blockage of the feeding channel, we do not need to vibrate the whole mold, but concentrate the vibrational energy in the feeding channel. Therefore, we proposed to use a waveguide rod to introduce the vibration to the blockage, where a small force can cause noticeable vibration efficiently. The waveguide rod-introducing method has extensively been used in high-intensity ultrasonic [27,28,29], but rarely in mechanical vibration. Therefore, the flexible targeted vibration-applying method could expand the application of mechanical vibration in the casting field.

In this work, mechanical vibration was introduced to the LPSC by the waveguide rod, and the shrinkage defects, microstructure, and mechanical tensile properties at room temperature were investigated to evaluate the effect of vibration and solidification pressure on the solidification feeding. Our objective was to propose an innovative method and idea for decreasing the shrinkage defects and increasing the mechanical properties of the LPSC castings made of Al-Cu-Mn-Ti alloy with a large solidification interval and to elaborate the mechanisms of them. The present study could provide a theoretical basis and technical support for the casting technology.

## 2. Materials and Methods

### 2.1. Chemical Analysis of Alloy

The chemical composition of Al-Cu-Mn-Ti alloy was analyzed using an optical emission spectrometer and presented in Table 1. Additionally, the liquidus and the solidus temperature were determined as 658 °C and 542 °C by differential scanning calorimetry, respectively. 

### 2.2. Casting Setup

The waveguide rod with an alcohol-based graphite coating material was embedded in the sand mold when modeling. This treatment cannot only reduce the influence on the transmission efficiency of the friction between the waveguide rod and the sand mold, but also diminish the impact of mold vibration on the LPSC equipment and its gas tightness. According to previous research, 45-steel has the best performance in the vibration transmission efficiency [30]; so, a 45-steel rod with a 10 mm diameter was used in the study.

**Figure 1:**
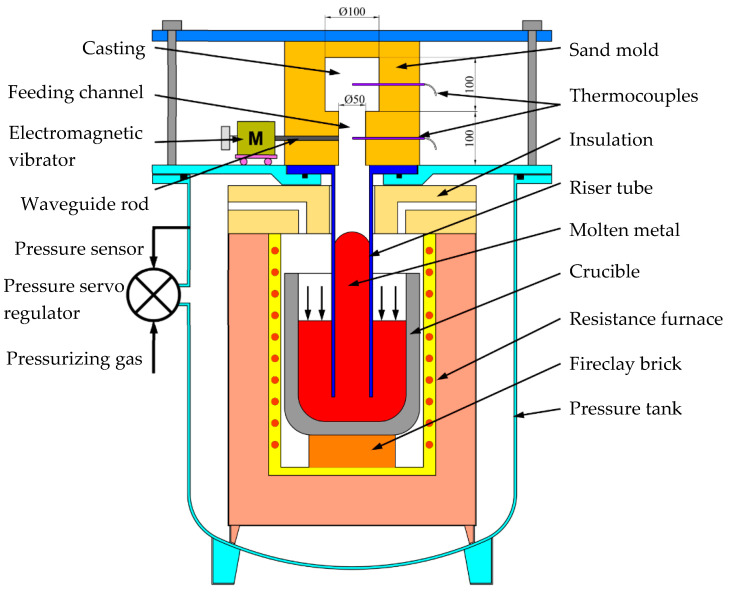
The LPSC system with vibration.

**Figure 2:**
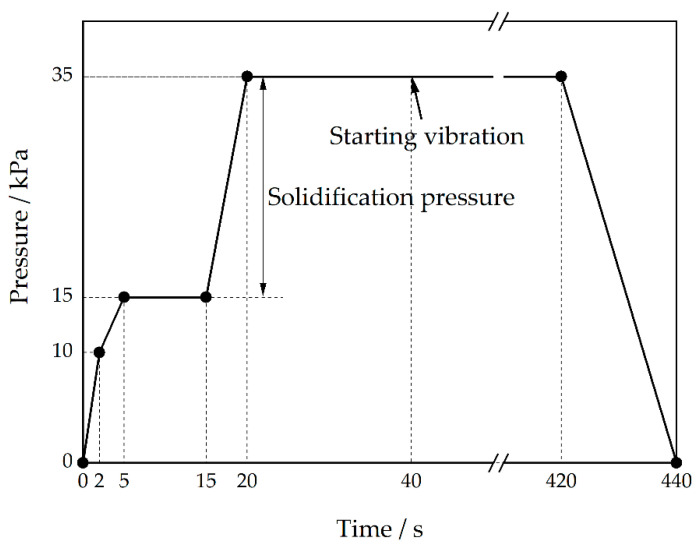
Pressure–time curve for pouring and solidification at 20 kPa solidification pressure.

As shown in Figure 1, Al-Cu-Mn-Ti alloy was smelted in a resistance furnace, degassed by the spinning rotor technology with injecting pure nitrogen at 700 °C, and kept at 680 °C after skimming. The liquid-level-increasing pressure and time, fill-in pressure and time, shelling time, pressure-increasing rate and time, and holding pressure and time in the LPSC process, as shown in Figure 2, were set in a high-precision air pressure control system. The vibration amplitude, converted to the voltage of the vibrator, and the vibration frequency required in the experiments were controlled by the electromagnetic vibrator. The melt temperature, monitored by a thermocouple, was stabilized at 680 °C for 5 min. Then, the riser tube, preheated to 700 °C, was inserted into the crucible. Next, a furan resin sand mold, assembled with the thermocouples in the center of the casting and feeding channel and the electromagnetic vibrator, was placed on the lid of the pressure tank, compressing the riser tube, of which the center corresponded to that of the inner gate, constricted by a press plate. Finally, starting the air pressure control system, the cavity was filled under the preset pressure. After 40 s filling, a solidified shell was formed in the casting, starting the vibrator to vibrate the casting.

### 2.3. Experimental Scheme

In order to compare the effects of vibration and solidification pressure on the feeding of the LPSC castings, experimental schemes were designed (see Table 2). Samples 1 and 2 solidified without vibration, but sample 2 was a twice the solidification pressure as sample 1. Samples 3 and 4 solidified with the same excitation force but different vibration frequencies, and their solidification pressures were also different. By measurement, the acceleration amplitudes of samples 3 and 4 were 0.5 and 11.5 m/s^2^, respectively, and the displacement amplitudes were 0.048 and 0.427 mm, respectively. 

### 2.4. Metallurgical Characterizations

**Figure 3:**
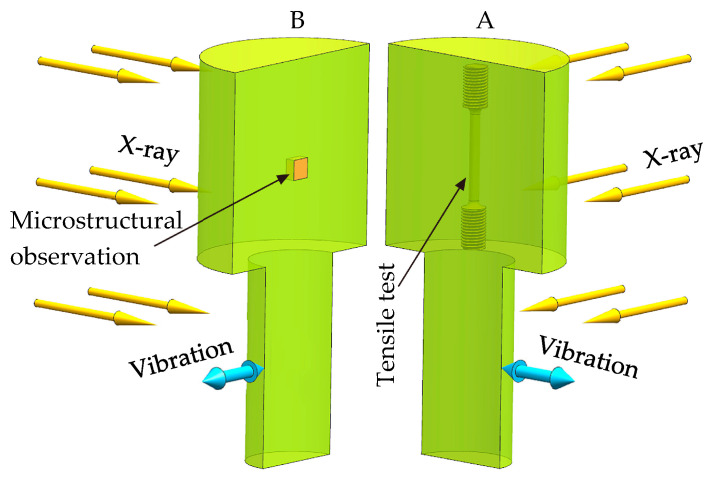
Locations for (**B**) microstructural observation and (**A**) tensile test and the direction for X-ray detections of each sample.

Firstly, the average densities of the cast samples were measured by an improved Archimedean method. Then, the samples were divided into two halves along the plane determined by the central axis and the vibration source, as shown in Figure 3. The distribution and size of the shrinkage defects were quantitatively analyzed by observing the sections and the X-ray detections. Afterwards, the metallographic samples were cut out in the center of the castings. Finally, a rod-shaped sample was cut at the position 6 mm away from the center of the casting, and a tensile test was carried out to obtain the mechanical properties.

#### 2.4.1. Density Measurement

The Archimedes principle was applied to calculate the average densities of the cast samples. After measuring the weight of each sample separately in air and water, using an electronic scale with a measuring accuracy of 0.01 g, the experimental density was calculated using the following equation:

(1) $$\rho_{\exp}=\frac{W_{a}}{W_{a}-W_{L}}\rho_{L}$$

where $\rho_{\exp}$ is the experimental density, $W_{a}$ is the weight in air, $W_{L}$ is the weight in water, and $\rho_{L}$ is the density of distilled water [31].

#### 2.4.2. Shrinkage Porosity Evaluation 

Firstly, the severity of shrinkage porosity was preliminarily judged by the section of samples. Then, the XYD-160/3 non-destructive X-ray detection machine was used to inspect the shrinkage porosities. The area of shrinkage defects, measured by ImageJ software (version V 1.8.0) on the X-ray films, was used to represent the size of shrinkage porosity.

#### 2.4.3. Microstructure Evaluation

The metallographic samples were polished using emery paper with different grits (400, 1000, 1500, 2000) and a micro-fiber cloth to achieve a mirror finish and then ultrasonically cleaned using ethanol. The samples were etched with 0.5% HF aqueous solution for 15 s followed by washing in distilled water and drying before the metallographic examination. The primary *α*-Al grain size was measured and analyzed using the linear intercept method in ImageJ software.

#### 2.4.4. Mechanical Property Test

Mechanical properties, such as the tensile strength and the elongation, of the cast samples were checked. The tensile samples were fabricated in accordance with GBT228-2002 (5 mm gauge diameter and 30 mm gauge length) for all the castings. The Instron-5500R tensile testing machine (Norwood, MA, USA) was used to perform the tensile test with a crosshead speed of 2.0 mm/min.

## 3. Results and Discussions

### 3.1. Harmonic Response of the Casting System

**Figure 4:**
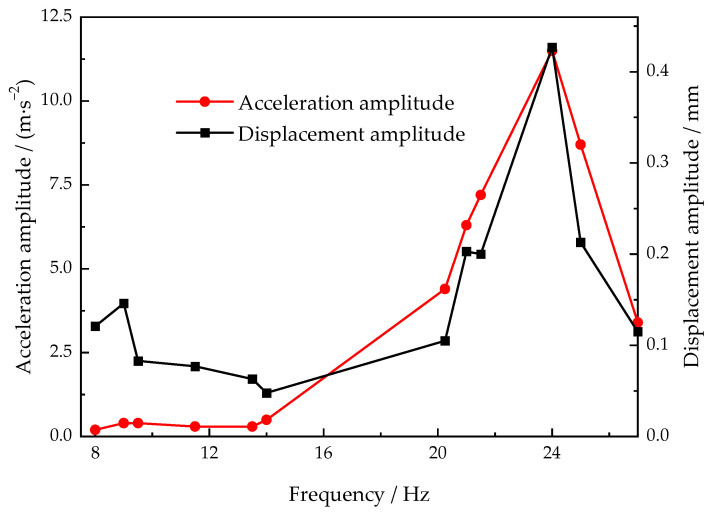
The amplitude–frequency response curves of acceleration and displacement of the LPSC system.

As the harmonic response analysis of the LPSC system is more accurate to reveal the relationship between the vibration parameters and the solidification characteristics, the amplitude–frequency response curves of acceleration and displacement before pressure relief were measured by a high-precision vibrometer, as shown in Figure 4. According to the definition, the natural frequency of each mode approximates the peak amplitude frequency of a system. The system may experience a resonant vibration if the excitation force frequency is near one of its natural frequencies. It was presented that the natural frequency of the LPSC system during solidification was 24 Hz in this study. The frequency of external excitation in sample 4 was equal to the natural frequency of the system. At this point, compared to the other frequencies such as the frequency of sample 3, the mechanical energy had the highest conversion efficiency in the casting, generating the largest displacement in the feeding channel if inputting the same excitation force.

### 3.2. Visual Observation

**Figure 5:**
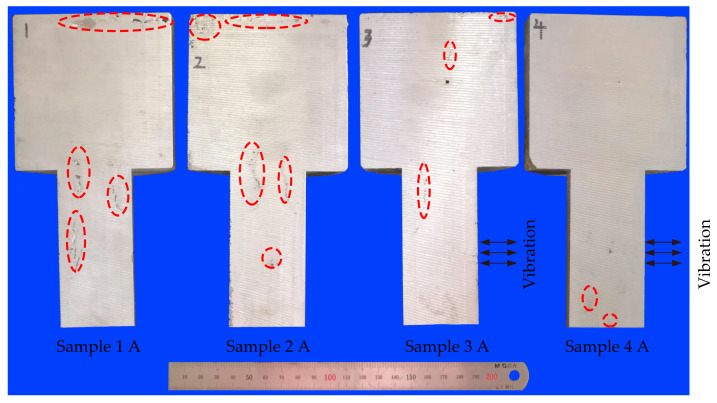
Appearances and porosity locations of samples.

There were serious shrinkage defects in samples 1 and 2, and the defects were concentrated in two locations (denoted by red, dashed ellipse in Figure 5): the top of the casting and the connection between the casting and the feeding channel. The formation of the top defects was attributed to the blocked feeding channel generated by the dendritic overlap in the late solidification stage, which increased the feeding resistance rapidly and impeded the liquid metal in the riser to feed the casting through the feeding channel. The shrinkage of the casting center, the final solidification part, was supplemented by the top melt because of the effect of gravity in the absence of external feeding, resulting in shrinkage defects concentrated at the top of the casting. The sand mold around the connection part was simultaneously subjected to the heat transferred from the feeding channel and the casting, resulting in poor heat dissipation, thus forming a physical hot spot. Meanwhile, in the filling process, the high-temperature liquid metal flowed to the casting through this part, thus heating the surrounding mold, forming a flow hot spot [32]. Therefore, this connection part was where the physical and the flow hot spot overlapped, which was difficult to feed and easy to form shrinkage defects. There was a large shrinkage cavity at the top of sample 1, but diffuse shrinkage porosities of sample 2. The location of the shrinkage defects in sample 3 was roughly the same as that in samples 1 and 2, but the defect area was significantly reduced. The casting part of sample 4 was completely free of shrinkage defects, but there were still defects in the lower feeding channel.

### 3.3. Shrinkage Defects and Density Variation

**Figure 6:**
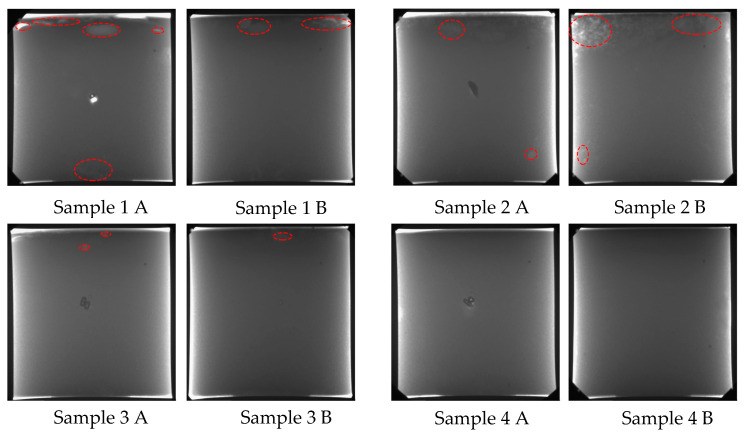
X-ray detection results of castings.

X-ray detection, as an effective way to locate the internal pores of castings, gives a quantitative description for the sizes and the distribution of shrinkage defects. As shown in Figure 6, the location of the defects (denoted by red, dashed ellipses) detected by X-ray was similar to that marked in the cross section shown in Figure 5, indicating that the shrinkage defects in the samples were mainly concentrated on the plane determined by the central axis and the vibration source. Vibration can eliminate shrinkage defects in castings, as shown in the casting of sample 4.

**Figure 7:**
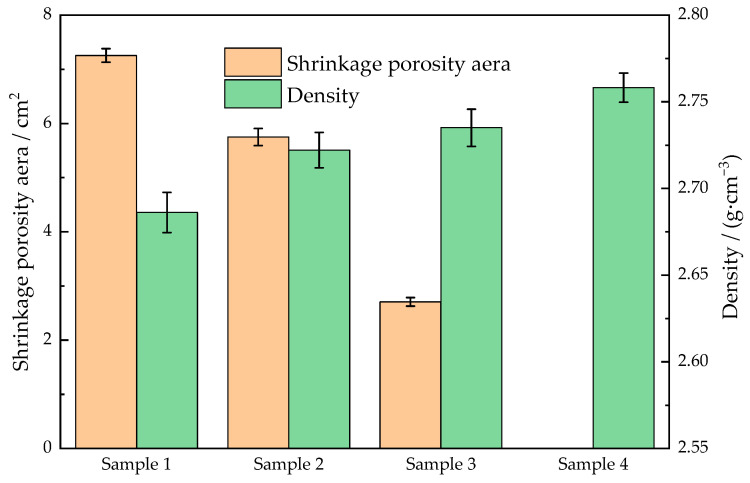
The shrinkage porosity areas and the average densities of samples.

As shown in Figure 7, the shrinkage defect areas of castings in samples 1–4 were 7.26, 5.75, 2.71, and 0 cm^2^, respectively, and the average densities were 2.686, 2.722, 2.735, and 2.758 g/cm^3^, respectively. The shrinkage defect areas in samples 2 and 3 were smaller and the densities were larger than that in sample 1, demonstrating that either increasing the solidification pressure or applying vibration in the LPSC process can reduce the shrinkage defects of castings and increase the densities. Comparing the defect areas between samples 1 and 2, the shrinkage defects were still serious even if the solidification pressure was doubled. From the comparison of defect areas between samples 1 and 3, the shrinkage defects were greatly improved with applying a small vibration even though the solidification pressure was low (20 kPa), indicating that only increasing the solidification pressure was not as effective as applying vibration to improve the feeding capacity. The same conclusion can be drawn by comparing the defect areas between samples 2 and 4. The results were consistent with Yadav’s experiments, which compared the shrinkage defects and microstructures of gravity castings of A308 alloy without and with 0–30 Hz mechanical vibration [20]. The shrinkage defects in sample 4 were eliminated, indicating that increasing the solidification pressure and applying vibration simultaneously maximized the feeding capacity of the LPSC. The above conclusions were also obtained by comparing the average densities of the samples, where sample 4 had the maximum density.

### 3.4. Microstructural Observation

**Figure 8:**
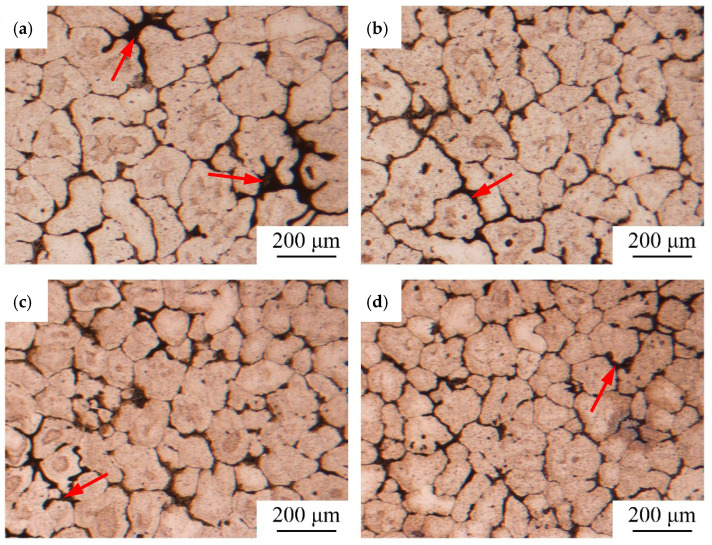
Optical micrographs of the center of castings: (**a**) sample 1; (**b**) sample 2; (**c**) sample 3; (**d**) sample 4.

As shown in Figure 8a, for sample 1 of low solidification pressure (20 kPa) without vibration, the shrinkage porosities in the interdendritic regions were the largest among all samples. By contrast, as shown in Figure 8b–d, for samples 2–4, the size of shrinkage porosities denoted by red arrows gradually reduced, indicating that either increasing the solidification pressure or applying vibration can reduce the shrinkage porosities in the LPSC castings.

As shown in Figure 8, the as-cast microstructural characterization of this alloy was a typical equiaxed grain morphology, as expected. The measured average α-Al grain sizes of the samples were 201, 187, 170, and 142 μm, respectively. As compared with sample 1, the grain sizes of samples 2–4 reduced by 7.2%, 15.2%, and 29.5%, respectively. The refinement of the mechanical vibration in the aluminum castings was observed generally by researchers in gravity castings [18,19,20,21,22,23,24]. The same refinement effect on the LPSC castings was firstly observed in our experiments. Combining the casting conditions, it was concluded that applying a small vibration (0.048 mm displacement amplitude) was more effective than increasing the solidification pressure (20 kPa to 40 kPa) at the refinement of grains. When the vibration frequency was increased to 24 Hz, the resonant frequency, the α-Al grain size was finest. 

Several methods in which vibration is applied to the molten metal for grain refinement have been reported. In these reports, the following refinement mechanisms were described: cavitation bubbles [33], the mechanical division of dendrites [34], the promotion of nucleation [35], and fragmentation by the melting of dendrites due to the convection of molten metal [36]. The published information for the effect of low-frequency vibration on cavitations is very limited [37]. Osawa et al. [38] reported that the mechanical division of the primary phase is difficult due to its rigidity. When starting the vibration, the nucleation has come to an end in the experiments. Therefore, the difference in grain size in the casting part may attribute to the change of the cooling rate, which is the key parameter for microstructures during solidification [39]. Since the interfacial heat transfer increases with the holding pressure in the LPSC process, accelerating the cooling rate of castings [40,41], the grains of sample 2 were smaller than that of sample 1. The cooling rate of the castings also increased with applying vibration [42], leading to the refinement of samples 3 and 4. The cooling rate was maximum at resonant frequency, showing significant refinement in the microstructure.

### 3.5. Mechanical Properties

**Figure 9:**
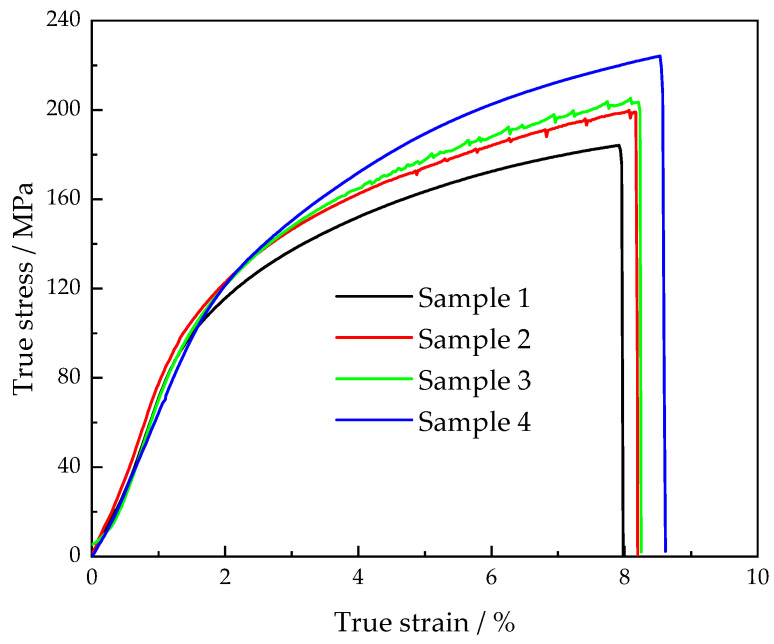
True stress–strain diagram of tensile test samples.

**Figure 10:**
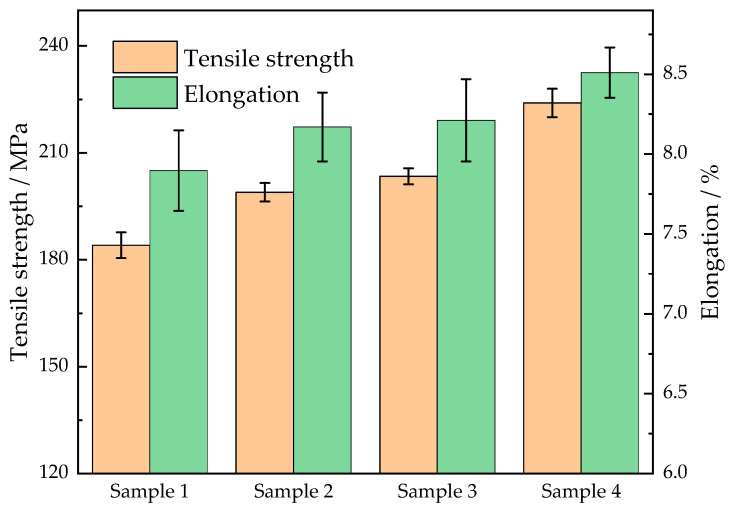
Comparison of mechanical properties of casting samples.

In this section, the influence of the solidification pressure and vibration on mechanical properties, ultimate tensile strength, and percentage elongation of the cast Al-Cu-Mn-Ti alloy under stationary and vibratory conditions is examined. The obtained results are summarized in Figure 9 and Figure 10. Figure 9 shows the true stress–strain diagram of the Al-Cu-Mn-Ti alloy under different conditions. The ultimate tensile strength of the samples was 184.1, 198.9, 203.4, and 224.0 MPa, respectively, and the percentage elongation was 7.9%, 8.1%, 8.2%, and 8.5%, respectively, as shown in Figure 10. Sample 4 under a high solidification pressure (40 kPa) and a large mechanical vibration (0.427 mm displacement amplitude) at the resonant frequency (24 Hz) demonstrated the highest tensile strength and elongation due to the microstructural refinement and the shrinkage defect elimination. The tensile strength and elongation of sample 4 were improved by 21.7% and 7.8%, respectively, as compared with sample 1, which solidified under low solidification pressure (20 kPa) without vibration. In terms of improving the mechanical properties of the LPSC castings, increasing the solidification pressure, such as with sample 2, was equivalent to using a small vibration, such as with sample 3.

### 3.6. Effect of Mechanical Vibration on the Solidification Feeding in the LPSC

The three main feeding mechanisms in the solidification of metals are mass feeding, interdendritic feeding, and burst feeding [43]. Mechanical vibration can influence them at different solidification stages.

#### 3.6.1. Mass Feeding

Mass feeding is the main mechanism to the casting before the feeding channel is blocked. Mass feeding denotes the movement of a slurry of solidified metal and residual liquid. This movement is arrested when the volume fraction of solid reaches anywhere between 0 and 50%, depending on the pressure differential driving the flow and depending on what percentage of dendrites are free from points of attachment to the wall of the casting. The important criterion to assess whether mass flow will occur is the ratio (casting section thickness)/(average grain diameter). In large sections, or where grains have been refined, the flow of the slurry can be easier to pass through the feeding channel [43]. According to the optical micrographs, shown in Figure 8, the average grain size was reduced after vibration treatment, which was conducive to mass feeding.

#### 3.6.2. Interdendritic Feeding

With the increasing percentage of the solid during solidification, the dendrites started to become a coherent network, which prevented the movement of grains. Interdendritic feeding is used to describe the flow of residual liquid through the pasty zone, which can be regarded as the seepage in porous media. The Al-Cu-Mn-Ti alloy melt is Bingham fluid [44], and its seepage can be expressed as

(2) $$q=\frac{K}{\eta }\left(P_{G}-P_{GT}\right)$$

where q is the seepage velocity, K denotes the permeability of the porous medium, η is the dynamic viscosity of fluid, and $P_{G}$ and $P_{\mathrm{GT}}$ are the instantaneous and the threshold pressure gradient along the flow direction, respectively.

In order to facilitate the description and calculation, we usually simplify the seepage channel into a circular capillary. The real seepage velocity can be corrected by multiplying a coefficient on this result. According to the simplification, the threshold pressure gradient can be expressed as

(3) $$P_{GT}=\frac{4}{3}\left(\frac{\tau_{s}}{R}\right)$$

where $\tau_{s}$ is the yield stress of Bingham fluid and R denotes the equivalent hydraulic radius of a seepage channel.

**Figure 11:**
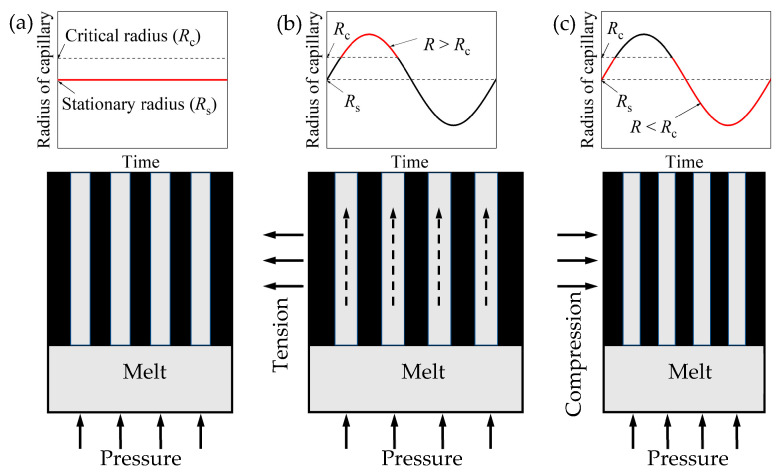
The process of vibration promoting interdendritic feeding: (**a**) stationary radius (*R*s) < critical radius (*R*c), interdendritic feeding is limited; (**b**) radius of capillary under vibration (*R*) > *R*c, interdendritic feeding is restarted; (**c**) *R* < *R*c, interdendritic feeding stops again.

According to Equation (2), the melt starts to flow only when the instantaneous pressure gradient is greater than the threshold. As the capillaries formed by dendrites overlapping become smaller with solidification, the threshold pressure gradient calculated by Equation (3) increases. If the instantaneous pressure gradient cannot overcome the threshold, the melt flow in the capillary stops completely, as shown in Figure 11a. Increasing the solidification pressure of low-pressure casting will restore the seepage flow at this moment; so, the shrinkage defects in sample 2 were reduced after increasing the holding pressure. With applying mechanical vibration, which is perpendicular to the feeding direction, the capillary diameter changes periodically due to vibration [45]. When the feeding channel experiences tensile stress, the diameter of the capillary increases, decreasing the threshold pressure gradient, restarting the seepage. As shown in Figure 11b, the red curve denotes the restarting period of seepage. On the contrary, the capillary diameter decreases when the feeding channel is in a compression-stressed condition, increasing the threshold pressure gradient, stopping the seepage. As shown in Figure 11c, the red curve denotes the repressed period of seepage. Therefore, there is a time section for the feeding channel to recover its feeding capacity in every vibration period, until the capillary diameter at the tension stage is smaller than the critical diameter due to solidification. Hence, compared with the stationary casting, the mechanical vibration promotes the interdendritic feeding.

#### 3.6.3. Burst Feeding

**Figure 12:**
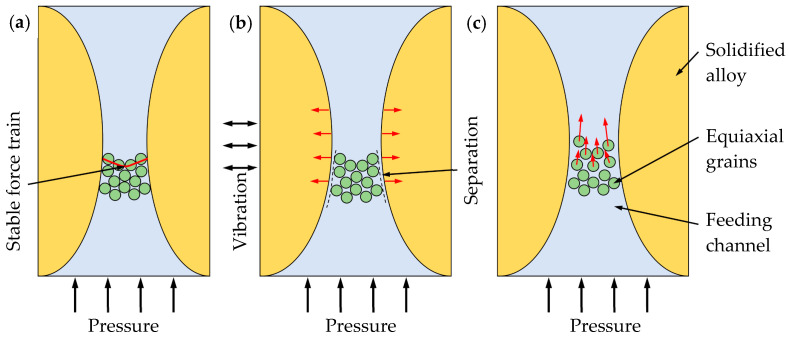
The process of vibration promoting burst feeding: (**a**) feeding was stopped by barrier with stable force chain; (**b**) the barrier and feeding channel wall was separated by vibration; (**c**) the force chain was destroyed and the barrier was broken, causing burst feeding.

Where hydrostatic tension is increasing in a poorly fed region of the casting, it seems reasonable to expect that any barrier might suddenly yield, as with a dam bursting, allowing feed metal to flood into the poorly fed region. This feed mechanism is burst feeding. As solidification proceeds, both the stress and the strength of the barrier will be increasing together, but at different rates. Failure will be expected if the stress grows to exceed the strength of the barrier. There is no doubt that increasing the feeding pressure is one of the ways to destroy the barrier, but by requiring a large feeding pressure. The vibration can also cause a barrier failure by changing the barrier structure rather than destroying the grains [14]. As shown in Figure 12a, when the feeding channel is blocked by grains, a stable force chain is formed between the grains and the feeding channel wall. Since the barrier is the grain accumulation caused by slurry flow, its bond strength with the feeding channel wall is much smaller than that of the channel wall itself formed by dendritic overlap. Hence, the barrier contacts and separates with the feeding channel wall periodically. At the separation, the channel wall no longer provides support for the grain chain and the force chain disappears, as shown in Figure 12b. Under the action of feeding pressure, the grains in the barrier move into the casting, leading to the collapse of the barrier, opening the feeding channel and forming the burst feeding, as shown in Figure 12c.

## 4. Conclusions

This study investigated the effect of mechanical vibration on the mechanical properties and solidification feeding during the LPSC process of Al-Cu-Mn-Ti alloy. The following conclusions were drawn.

- Applying a suitable vibration, such as the resonant vibration, was more effective than increasing solidification pressure in improving solidification feeding of the LPSC, while the most effective method was applying both simultaneously, which eliminated the shrinkage defects and increased the density by 2.7%.
- By applying resonant vibration and increasing the solidification pressure simultaneously, metallurgical features such as the average values of the primary *α*-Al grain size and average density reduced by 29.5% and increased by 2.7%, respectively; the mechanical properties, such as ultimate tensile strength and percentage elongation increased by 21.7% and 7.8%, respectively, as compared with low solidification pressure and stationary casting.
- Mechanical vibration was conducive to mass feeding by refining the primary α-Al grains, to interdendritic feeding by reducing the threshold pressure gradient, and to burst feeding by collapsing the barrier.

## Figures and Tables

**Table 1:** Chemical composition of Al-Cu-Mn-Ti Alloy (wt.%).

Cu	Mn	Ti	Zr	B	Cr	V	Al
5.0	0.4	0.2	0.15	0.03	0.15	0.08	Bal.

**Table 2:** Casting scheme.

Castings	Solidification Pressure (kPa)	W/wo Vibration	Frequency (Hz)	Acceleration Amplitude (m/s^2^)	Displacement Amplitude (mm)
Sample 1	20	w/o	—	—	—
Sample 2	40	w/o	—	—	—
Sample 3	20	w/	14	0.5	0.048
Sample 4	40	w/	24	11.5	0.427

## Data Availability

The data presented in this study are available on request from the corresponding author.
